# Hypoglycemic Effect of Astaxanthin Oil–Fish Oil Complex in Mice With Type 2 Diabetes

**DOI:** 10.1002/fsn3.72410

**Published:** 2026-10-07

**Authors:** Zhichao Lin, Jianlin He, Xiaomin Xi, Yiping Zhang, Qingqing Le, Yaqi Guo, Hua Fang, Wenhui Jin, Hui Chen, Sijin Chen, Weizhu Chen

**Affiliations:** ^1^ Third Institute of Oceanography Ministry of Natural Resource Xiamen Fujian People's Republic of China; ^2^ School of Advance Manufacturing/School of Ocean Fuzhou University Jinjiang Fujian People's Republic of China; ^3^ Xiamen Ocean Vocational College Xiamen Fujian P. R. China

**Keywords:** adjuvant therapy, Astaxanthin, fish oil, glucose metabolism, insulin resistance, oxidative stress

## Abstract

Diabetes mellitus (DM), a chronic metabolic disorder, is characterized by disrupted glucose homeostasis, and its escalating global prevalence underscores the critical need for safe dietary interventions. This study investigated the hypoglycemic and hepatoprotective potential of a novel formulation combining astaxanthin oil (AO) and fish oil (FO) as an adjuvant nutritional strategy. The efficacy of the AO–FO complex was evaluated using the insulin‐resistant HepG2 cell model in vitro and high‐fat diet (HFD)‐fed insulin‐resistant mice in vivo. While individual treatments with AO and FO improved basic insulin sensitivity, their co‐administration exhibited enhanced efficacy in promoting cellular glucose consumption. Notably, the combined formulation was associated with changes in key glucose metabolism‐related indices, including glucose‐6‐phosphate, pyruvate kinase, hexokinase, and significantly mitigated oxidative stress markers. In vivo, the AO‐FO complex showed greater effects on decreasing fasting blood glucose, improving oral glucose tolerance, and ameliorating systemic dyslipidemia compared to singular oil treatments. Furthermore, histopathological evaluations revealed a marked attenuation of hepatic steatosis and pancreatic islet damage following the combined treatment. Collectively, these findings support the potential combined benefits of astaxanthin and omega‐3 polyunsaturated fatty acids for improving glucose homeostasis, suggesting that the AO–FO complex may be a candidate functional food for DM management.

## Graphical abstract

This study demonstrates that an astaxanthin oil–fish oil complex exerts synergistic hypoglycemic and hepatoprotective effects. It enhances glucose metabolism and relieves oxidative stress in insulin‐resistant HepG2 cells. In high‐fat diet mice, the complex reduces hyperglycemia, improves lipid profiles, and attenuates hepatic steatosis and pancreatic islet injury. The AO–FO complex holds potential as a functional food for diabetes mellitus.

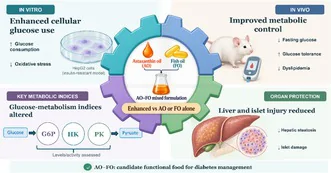

## 1 Introduction

Diabetes mellitus (DM), a chronic metabolic disorder, is characterized by sustained hyperglycemia due to dysregulated insulin activity and/or insulin production (Jing et al. 2020). At present, DM affects 382 million people worldwide, with a predicted increase to 592 million by 2035. DM not only substantially decreases patients' quality of life, but also elevates mortality and their economic burden. Its chronic nature and complications, along with the economic burden on the patient and their family members, have made this condition a major global health concern (Saeedi et al. 2019; Zhou et al. 2024). DM can be primarily categorized as Type 1 diabetes mellitus, Type 2 diabetes mellitus (T2DM), gestational diabetes, or other types due to tumors, pancreatitis, and other conditions. T2DM accounts for over 90% of diagnosed cases. It exhibits typical features of insulin resistance (IR) alongside relative insulin deficiency, leading to persistent hyperglycemia and multi‐organ complications, including nephropathy, retinopathy, and cardiovascular disease (Galicia‐Garcia et al. 2020; Ojo et al. 2023). Rapid urbanization and lifestyle transitions, including high‐calorie diets and physical inactivity, have significantly contributed to the increasing incidence of T2DM, which is now affecting younger populations at an alarming rate. This trend underscores the urgent need to develop effective and safe strategies for alleviating metabolic dysfunction while preventing diabetes‐related complications. At present, insulin and oral hypoglycemic agents, including thiazolidinediones, biguanides, and sulfonylureas, are the major treatment modalities for T2DM. However, these agents may cause notable adverse effects, including gastrointestinal disturbance or weight gain. Therefore, identifying a safe and efficacious dietary supplement may offer a promising approach to adjuvant therapy for diabetes management and metabolic disorder prevention, thereby addressing current limitations of conventional pharmacological interventions (Lima et al. 2022). Therefore, exploring natural and safe adjuvant therapies has become an important research area (Arroyave et al. 2020; Wang et al. 2017).

Astaxanthin (AXN) is a potent lipophilic carotenoid antioxidant. This nutraceutical has garnered considerable scientific interest since the 1990s owing to its superior bioactivity and broad therapeutic potential (Shokri‐Mashhadi et al. 2025). Natural AXN, as a clinically relevant nutraceutical, exhibits multifaceted pharmacological properties, including potent antioxidant, anti‐inflammatory, antiaging, and organ protective effects, particularly in cardiovascular, neurological, gastrointestinal, and renal systems (Khadilkar et al. 2023; Chen et al. 2020). Emerging research suggests its promising role in metabolic regulation, with abilities to enhance insulin sensitivity, improve glucose metabolism, and modulate lipid profiles. Growing evidence suggests that AXN exerts a beneficial effect on DM, which is achieved via multiple targets and pathways across various tissues and organs. In particular, AXN decreases blood glucose levels via three primary mechanisms: preserving pancreatic β‐cell function, alleviating IR, while enhancing insulin production. Notably, many studies have revealed the protective effects of AXN against macrovascular (peripheral arterial disease, stroke, coronary artery disease) and microvascular (diabetic nephropathy, retinopathy, neurological damage) complications of DM, with research focusing on the underlying cellular and molecular mechanisms. These findings position AXN as a valuable candidate for integrated diabetes management strategies (Zhang et al. 2021; Roohbakhsh et al. 2017; Gowd et al. 2021). AXN is biosynthesized in various organisms, such as the microalga *Haematococcus pluvialis* ( *H. pluvialis* ), fungi, marine invertebrates (krill and copepods), certain plants, and avian species (flamingos and quails). At present, *H. pluvialis* ‐derived AXN is the only approved source for human consumption. Owing to its well‐documented therapeutic benefits, it is utilized in aquaculture, nutraceutical, and healthcare industries (Dewati et al. 2023).

Fish oil (FO) is rich in omega‐3 polyunsaturated fatty acids (PUFAs), particularly docosahexaenoic acid (DHA) and eicosapentaenoic acid (EPA), which are known for broad‐spectrum health effects (Das et al. 2024). Omega‐3 PUFAs exhibit well‐documented anti‐inflammatory properties, favorable lipid‐modulating effects, and enhanced insulin sensitivity; these properties collectively contribute to improved glycemic control and reduced cardiovascular risk among those developing metabolic disorders (Karsli 2021). The benefits of supplementing omega‐3 PUFA in DM are mediated by two primary mechanisms: anti‐inflammatory activity and autoimmunity suppression (Iwase et al. 2015). Several reports have found that omega‐3 PUFAs exert protective effects against DM pathogenesis (Titisari et al. 2023). Notably, clinical evidence suggests that FO supplementation can mitigate diabetic complications, including nephropathy and retinopathy, while also inducing differential changes in lipidomic profiles and gut microbiota composition in T2DM (Lu et al. 2025). Emerging research further suggests that the FO and vitamin D combination may be a promising approach to preventing the onset and development of chronic kidney disease in individuals with T2DM (de Boer et al. 2019). Furthermore, preclinical studies have revealed that FO not only ameliorates cognitive and learning deficits in streptozotocin‐induced mice with diabetes but also protects against age‐related cognitive decline and depressive‐like behaviors in prediabetic rat models (Souza et al. 2020; Paula Farias Waltrick et al. 2022). Taken together, the above findings emphasize the dual effects of FO on metabolic regulation and neuroprotection. Moreover, FO enhances AXN bioavailability, with their co‐administration exhibiting synergistic effects, potentially amplifying their individual therapeutic benefits (Barros et al. 2012; Xia et al. 2024). The antioxidant properties of AXN can complement the anti‐inflammatory and metabolic regulatory effects of FO, thereby enhancing blood glucose control and decreasing complications (Saw et al. 2013). This combination strategy presents a compelling approach for improving metabolic health and mitigating diabetic complications.

However, the hypoglycemic effects of the combined supplementation of AXN and FO remain uninvestigated. Therefore, developing functional foods with these bioactive ingredients for adjuvant hypoglycemic therapy holds considerable potential for future applications. In this study, we used astaxanthin oil (AO) as an AXN source and combined it with FO to develop a novel adjuvant hypoglycemic product: the AO–FO complex. We then conducted comprehensive experiments using in vitro (insulin‐resistant HepG2 cells) and in vivo models (Type 2 diabetic mice) and evaluated this complex's potential to improve IR‐related indices, enhance glucose consumption, and reduce hyperglycemia, with comparisons with AO and FO alone. These findings provide new insights into managing diabetes and its associated complications, offering an approach to more effectively and safely treat T2DM.

## 2 Materials and Methods

### 2.1 Chemicals and Materials

AO (containing 10% AXN) was purchased from Yunnan Aierfa Biotechnology. FO (containing 35% EPA‐EE and 25% DHA‐EE) was purchased from Zhoushan Xinuojia Bioengineering. Dulbecco's modified Eagle medium (DMEM) was acquired in Xiamen Junshenghong Trading Co. Ltd. Fetal bovine serum (FBS) was purchased in Nanjing Hemiao Biotechnology Co. Ltd. Both the high‐fat diet (HFD) and control diet (containing 60% and 10% kcal from fat separately) were acquired in Jiangsu Synergy Pharmaceutical Bioengineering. The superoxide dismutase (SOD), malondialdehyde (MDA), catalase (CAT), glucose‐6‐phosphate (G6P), pyruvate kinase (PK), hexokinase (HK) activity, and glycogen synthesis assay kits were acquired in Nanjing Jiancheng Bioengineering Institute.

### 2.2 Cell Experiments

#### 2.2.1 Insulin‐Resistant HepG2 Cell (IR‐HepG2) Model Construction

HepG2 cells (Chinese Academy of Sciences Shanghai Cell Bank) were cultured in DMEM containing 25 mM glucose and 10% FBS at 37°C with 5% CO_2_. To establish the cell model, logarithmic‐growth‐phase HepG2 cells underwent 5 min of centrifugation (1000 rpm), resuspension, and inoculation into the 96‐well plate (10,000 cells/well) before incubation at 37°C (Tanaka‐Yachi et al. 2022). Cells were divided into two groups: normal and IR‐HepG2 model groups. The normal group was cultured in DMEM that contained 25 mM glucose, whereas the IR‐HepG2 model group was cultured in DMEM including 30 mM glucose alongside insulin at concentrations of 1 × 10^−8^, 1 × 10^−7^, 1 × 10^−6^, 1 × 10^−5^, and 1 × 10^−4^ mol/L. Both groups received medium supplemented with 1% FBS. Six replicate wells were used for each condition. Following incubation for 48 h, culture medium was discarded. Then, cells in both groups were all cultured for additional 24 h within serum‐free, phenol‐red‐free DMEM that contained 25 mM glucose and 10^−9^ mol/L insulin.

To determine cell viability, HepG2 cells (10,000/well) were seeded into a 96‐well plate and incubated for 24 h at 37°C with 5% CO_2_. Fresh medium containing the test compounds (AO and FO) at varying doses (1, 50, 100, 200 μg/mL) was used to replace the original medium. Six replicates were set for each group. After a 24‐h incubation period, cell viability was measured using a Cell Counting Kit‐8 (CCK‐8) assay.

#### 2.2.2 Grouping and Drug Administration

The experiment was performed in the normal, model, positive, AO, FO, low‐concentration (complex‐L), medium‐concentration (complex‐M), and high‐concentration (complex‐H) groups. The normal group was supplemented with DMEM that contained 25 mM glucose alongside 1% FBS, whereas those remaining groups were maintained within DMEM including 30 mM glucose, 1 × 10^−7^ mol/L insulin, and 1% FBS for 48 h to induce IR in cells. After 48 h, the culture medium was discarded. Then, both groups were cultured within serum‐free, phenol‐red‐free DMEM encompassing 25 mM glucose and 10^−9^ mol/L insulin, and the model group also received 10 mmol/L rosiglitazone treatment (Matsunaga et al. 2012; Moon et al. 2012). The AO and FO groups were treated with AO (2.5, 5, 10, 20, 50, 100 μg/mL) and FO (5, 10, 20, 40, 60, 80 μg/mL) for a 24‐h period, respectively. The complex‐L, complex‐M, and complex‐H groups received 24 h of AO–FO complex treatment: 5 μg/mL AO +60 μg/mL FO, 10 μg/mL AO +120 μg/mL FO, and 50 μg/mL AO +600 μg/mL FO, separately. The concentrations of AO, FO, and the AO–FO complex were selected based on preliminary dose‐screening experiments.

#### 2.2.3 Glucose Consumption Assay

Cell culture supernatant was collected, and glucose concentrations were determined with the glucose content assay kit (BC2500, Solarbio, Beijing, China) following specific protocols. The glucose concentrations in all samples (mmol/L) were calculated by dividing sample optical density (OD) at 505 nm by that of the standard solution and multiplying the results by 1 mmol/L. Cellular glucose consumption was then calculated by subtracting glucose content remaining in cell supernatant from that of original culture medium. Results were expressed as absolute glucose consumption (mmol/L) and were not normalized to cell viability, cell number, or protein content.

#### 2.2.4 Detection of HK, PK, G6P Activity, and Glycogen

Logarithmic‐growth phase HepG2 cells (300,000/well) were seeded in 6‐well plates before 24 h of incubation under 37°C with 5% CO_2_. The original medium was discarded after an overnight culture, then cells were rinsed once by PBS. Freshly prepared 1 × 10^−7^ insulin and 1% FBS‐supplemented high‐glucose medium (30 mmol/L) were introduced into every well, followed by 48 h of culture. Cells were then treated with samples from different groups as described in section 2.2.3, collected, and lysed. The cell grinding fluid was used to measure HK, PK, G6P activity, and glycogen.

#### 2.2.5 Measurement of Antioxidant Indices

Logarithmic‐growth phase HepG2 cells (300,000/well) were inoculated into 6‐well plates, followed by 24 h of incubation under 37°C in a 5% CO_2_ incubator. After medium removal following an overnight culture, cells were rinsed once with PBS. Freshly prepared 1 × 10^−7^ insulin and 1% FBS‐supplemented high‐glucose medium (30 mmol/L) were introduced into every well for a 48‐h culture period. Cells were then treated with samples from different groups as described in section 2.2.3, followed by washing twice with pre‐cooled PBS. Cells were then collected on ice, and measurements were performed according to the manufacturers' manuals of the SOD, MDA, and CAT detection kits.

### 2.3 Animal Experiments

The animal experimental protocols were approved by the Institutional Animal Care and Use Committee of the Third Institute of Oceanography (TI0‐IACUC‐02‐2025‐01‐17). Animal treatment was conducted according to the Guide for the Care and Use of Laboratory Animals (National Research Council, USA). Seventy 8‐week‐old male C57BL/6J SPF‐grade mice (weights, 18 ± 2 g; Guangdong Yaokang Biotechnology) were used in this study. After 1 week of acclimatization, the mice were randomized into the following groups: normal (*n* = 10, control diet) and HFD (*n* = 60, fed a HFD for 2 months to establish an insulin‐resistant hyperglycemic mouse model) groups.

After establishing the model, insulin‐resistant hyperglycemic mice were grouped and fed as follows: (1) model group: fed an HFD and given gavage of corn oil; (2) positive group: given an HFD and gavage of a metformin solution prepared using 0.5% carboxymethylcellulose sodium at 200 mg/kg body weight (BW); (3) AO group: fed an HFD and gavaged with an AO solution prepared using corn oil at 10 mg/kg BW; (4) FO group: fed an HFD and gavaged with a FO solution prepared using corn oil at 120 mg/kg BW; (5) low‐dose AO–FO complex group (complex‐L): fed an HFD and gavaged with the AO–FO complex prepared using corn oil at a combined dose of AO + FO (10 + 120) mg/kg BW; and (6) high‐dose AO–FO complex group (complex‐H): fed an HFD and gavaged with the AO‐FO complex prepared using corn oil at a combined dose of AO + FO (40 + 480) mg/kg BW. Each group received the corresponding dose once daily via oral gavage for 4 weeks. Animals in the normal group received a control diet simultaneously. Food intake, body weight, and fasting blood glucose levels were measured, and an oral glucose tolerance test (OGTT) was also carried out.

Following a 4‐week drug administration process, the animals received a 12‐h fasting period for food before anesthesia induction. They were allowed to take water unrestrictedly. Whole blood was collected via orbital bleeding before cervical dislocation, clotted under ambient temperature (25°C) for 2 h, and centrifuged (3500 rpm, 10 min, 4°C) to obtain serum. Serum samples were preserved under −80°C for subsequent biochemical analyses. The liver and pancreas were carefully dissected, their weights were measured, and they were immersed within 10% neutral buffered formalin solution prior to further histological analysis.

#### 2.3.1 Measurement of Fasting Blood Glucose Level

After successfully establishing the T2DM mouse model, animals received a 4‐h fasting period for food and could take water unrestrictedly. Thereafter, tail vein blood was harvested by cutting the tail to measure fasting blood glucose, which was considered the initial blood glucose level. Following drug treatment for 4 weeks, the animals received another 4‐h fasting period for food, and were allowed to take water unrestrictedly. Their fasting blood glucose levels were measured using the same method (by collecting blood from the tail vein by cutting the tail).

#### 2.3.2 OGTT in Mice

After 4 weeks of oral gavage treatment, all animals were subjected to a 4‐h fasting period for food, and they could take water freely. Afterward, the initial fasting blood glucose levels (0 min) of each mouse group were measured as baseline data. Subsequently, mice in each group were orally administered their corresponding test compounds. After 20 min, all mice received a single oral gavage of glucose solution (2 g/kg BW). Tail vein blood was harvested at 30, 60, 90, and 120 min following glucose administration to measure blood glucose levels.

#### 2.3.3 Detection of Serum Biochemical Indices

An automatic biochemistry analyzer was used to measure biochemical indices using the collected serum samples. The indices measured were aspartate aminotransferase (AST), alanine aminotransferase (ALT), high‐density lipoprotein cholesterol (HDL‐C), low‐density lipoprotein cholesterol (LDL‐C), triglyceride (TG), and total cholesterol (TC). All biochemical kits were acquired in Nanjing Jiancheng Bioengineering Institute.

#### 2.3.4 Oxidative Stress Indicator Measurement Within Liver Tissue

Liver tissues from each treatment group were retrieved from the −80°C refrigerator, and approximately 0.1 g was weighed. Pre‐cooled physiological saline was added at a weight‐to‐volume (g)‐to‐volume (mL) ratio of 1:9. The resultant mixed sample was thoroughly homogenized before centrifugation (3500 rpm, 10 min, 4°C) to harvest supernatants. SOD, glutathione, CAT, and MDA contents within liver tissues were determined by biochemical kits (Nanjing Jiancheng Bioengineering Institute).

### 2.4 Statistical Analysis

All results are presented as mean ± standard deviation (SD). Differences among groups were analyzed by ANOVA with multiple comparisons. GraphPad Prism 9.5 was used to conduct statistical analyses and plot figures. *p* < 0.05 indicated statistical significance, whereas *p* < 0.01 indicated high statistical significance.

## 3 Results and Discussion

### 3.1 Cell Experiments

#### 3.1.1 Cell Viability Determination

Cytotoxicity of AO, FO, and the AO–FO complex was evaluated using the CCK‐8 method (Figure 1). We noted that AO and FO exhibited no evident cytotoxicity against HepG2 cells within this concentration range (Boesch‐Saadatmandi et al. 2011).

**FIGURE 1:**
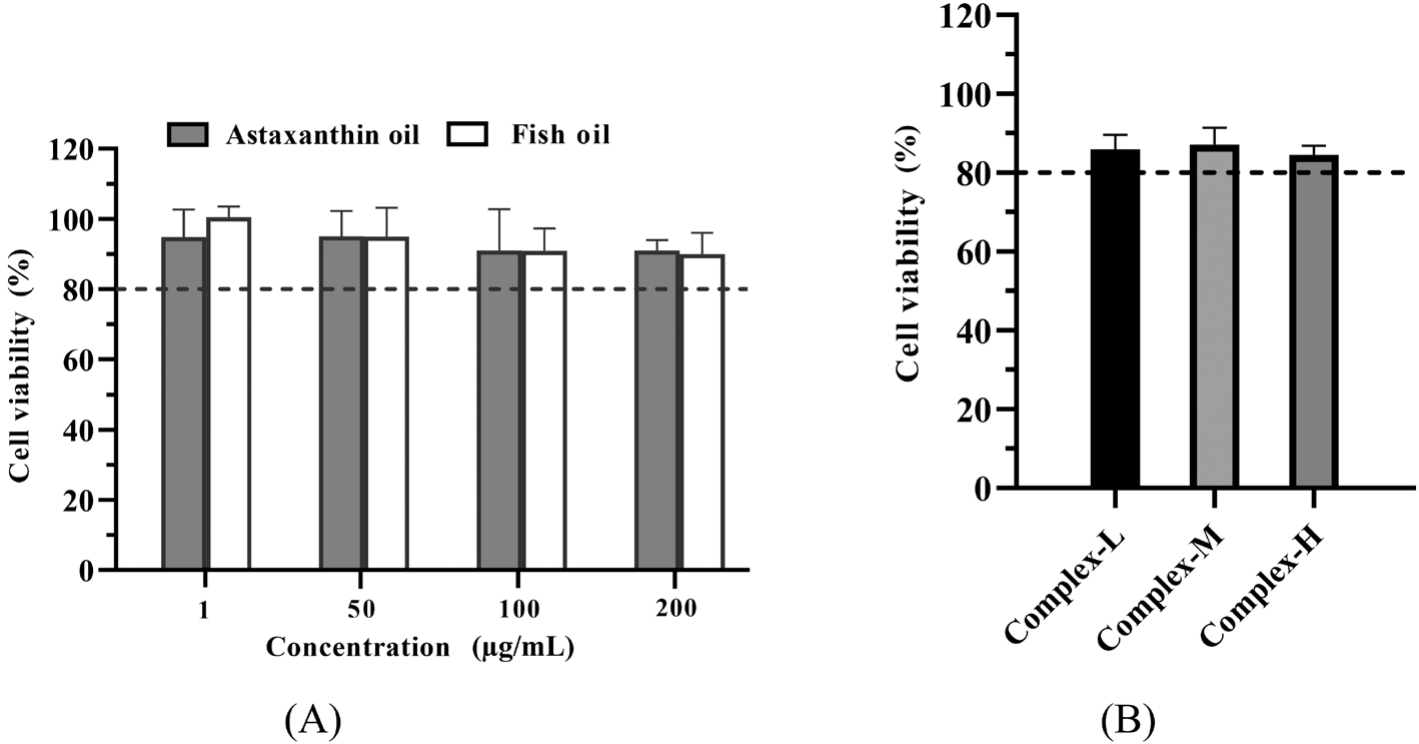
Effect of different concentrations of the samples on cell viability: (A) AO and FO and (B) AO–FO complex.

#### 3.1.2 Establishment of IR‐HepG2 Cell Model

As shown in Figure 2, after 48 h of exposure to 10^−7^ mol/L insulin, glucose consumption was significantly inhibited (*p* < 0.0001), with the lowest glucose consumption level. Therefore, insulin at 10^−7^ mol/L was chosen for later experiments to establish the IR‐HepG2 model. Because IR‐HepG2 model validation was based primarily on reduced glucose consumption under the selected induction condition and did not include direct measurements of insulin signaling or glucose transport, the model should be interpreted as an operational in vitro model of IR.

**FIGURE 2:**
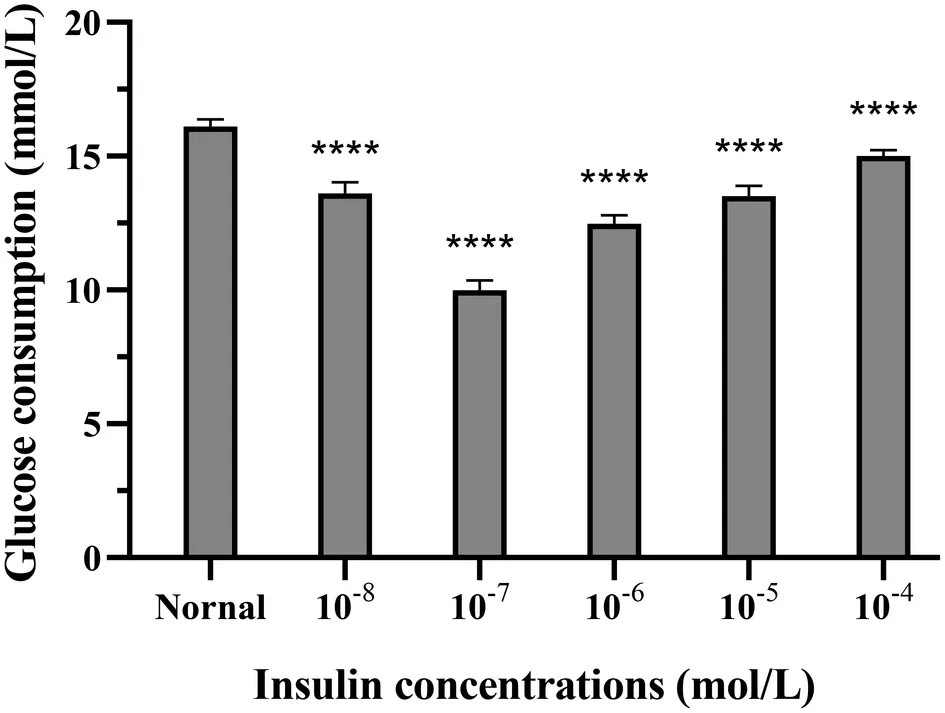
Effect of different insulin concentrations on glucose consumption in IR‐HepG2 cells. ****p < 0.0001 compared with the normal group.

#### 3.1.3 Detection of Glucose Consumption

Cellular glucose consumption is commonly used as an indicator of IR (Bhakta et al. 2017). The effects of AO and FO on IR‐HepG2 cells were evaluated by measuring glucose consumption. As illustrated in Figure 3, both AO and FO significantly and dose‐dependently increased glucose consumption. Furthermore, the AO and FO combination at a 1:12 ratio exhibited enhanced efficacy. For example, glucose consumption was enhanced by 94.24% in the complex‐H relative to the model groups, significantly outperforming individual treatments. These findings indicate that combined AO and FO treatment enhanced cellular glucose consumption under the experimental conditions.

**FIGURE 3:**
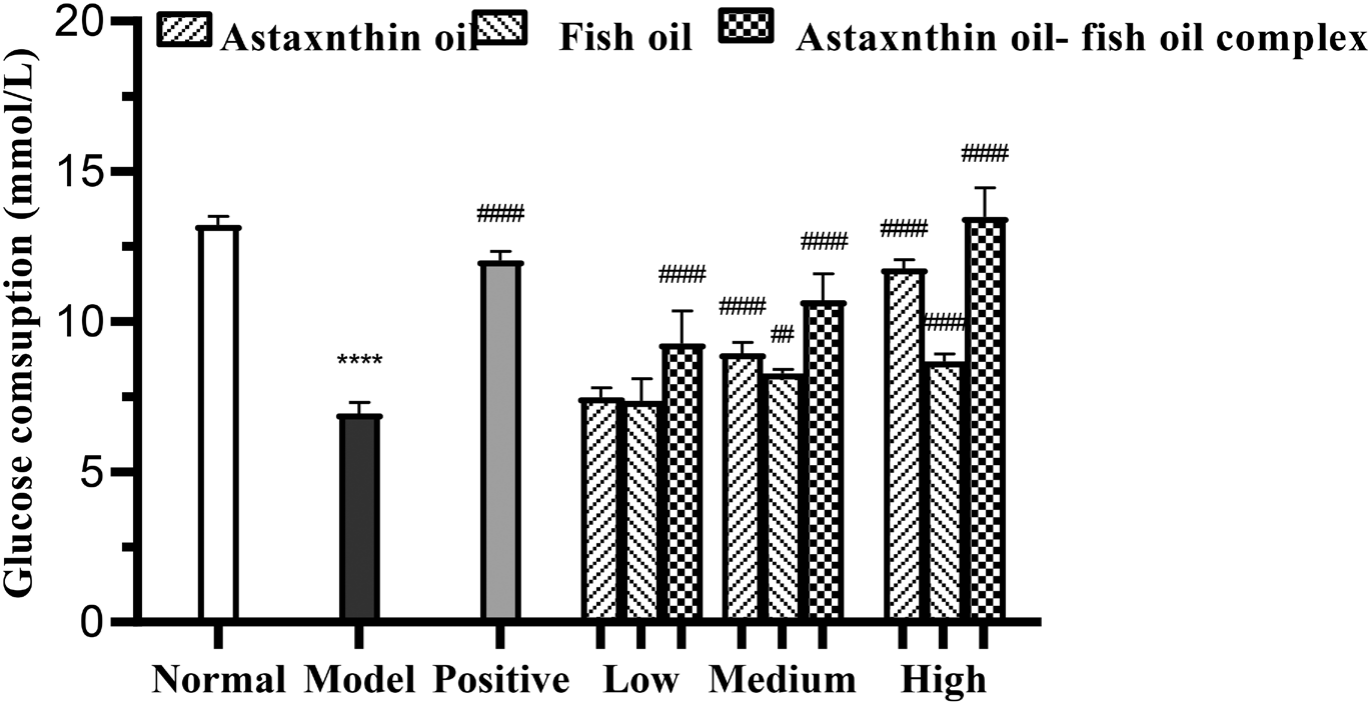
Effect of test samples on glucose consumption in IR‐HepG2 cells. ****p < 0.0001 compared with the normal group, and ##*p* < 0.01, ####*p* < 0.0001 compared with the model group.

#### 3.1.4 HK Activity Detection

As a glycolytic enzyme, HK catalyzes glucose phosphorylation to glucose‐6‐phosphate, an initial process of intracellular glucose metabolism (Zhang et al. 2020). The HK activity within IR‐HepG2 cells was measured after treatment with the different groups. As shown in Figure 4, the model group had a significantly decreased HK activity compared with the normal group, whereas HK activity in the positive control group was markedly higher than that in the model group. Neither AO nor FO alone altered HK activity. However, HK activity was significantly increased dose‐dependently in both complex‐M and complex‐H groups relative to the model group.

**FIGURE 4:**
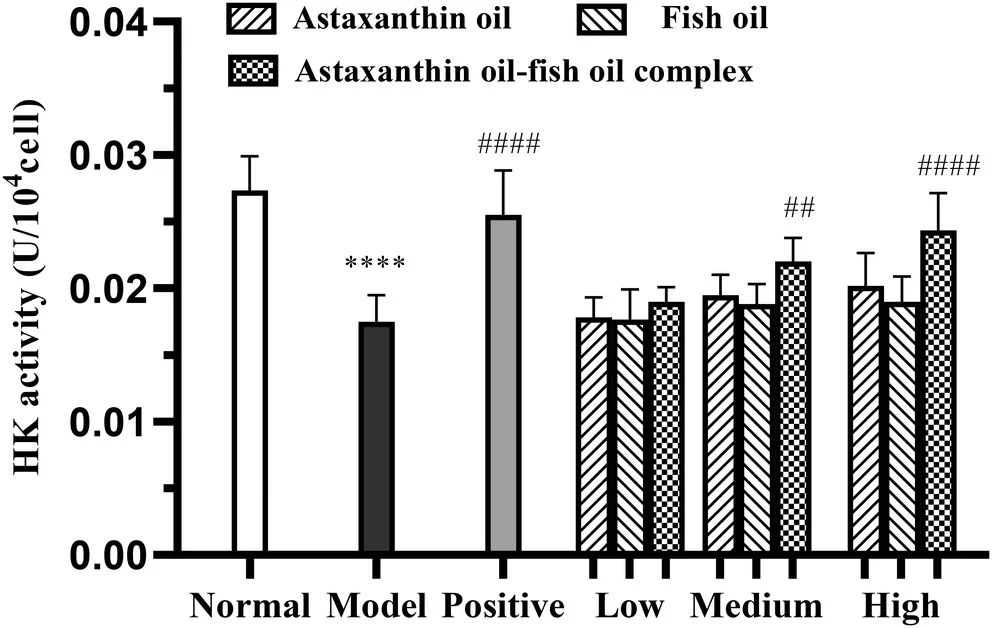
Effect of the test samples on HK activity in IR‐HepG2 cells. ****p < 0.0001 compared with the normal group, and ##*p* < 0.01 and ####*p* < 0.0001 compared with the model group.

#### 3.1.5 Detection of PK Activity

PK regulates glycolysis by catalyzing the conversion of phosphoenolpyryvate to pyruvate (Wang et al. 2024). As illustrated in Figure 5, PK activity was markedly suppressed in the model versus normal groups, indicating disrupted glucose metabolic function in IR‐HepG2 cells. Treatment with AO, FO, and their combinations increased PK activity to varying degrees. Among these treatments, complex‐M and complex‐H exhibited the most pronounced restoration, with significantly higher PK activity than the model group and higher activity than the AO‐ and FO‐treated groups. These findings indicate that the AO–FO complex was more effective than either component alone in modulating PK activity under the experimental conditions. Further studies are needed to determine whether these changes are associated with alterations in glucose uptake, insulin signaling, or other metabolic pathways.

**FIGURE 5:**
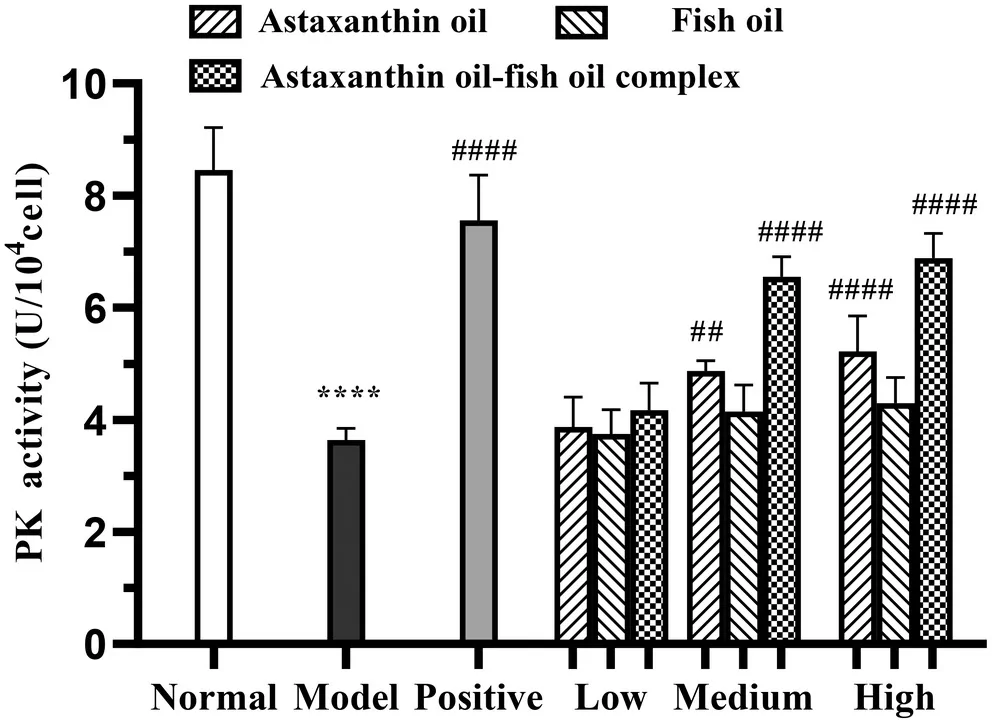
Effect of the test samples on PK activity in IR‐HepG2 cells. ****p < 0.0001 compared with the normal group, and ##*p* < 0.01 and ####*p* < 0.0001 compared with the model group.

#### 3.1.6 Detection of G6P Activity

G6P‐related activity is associated with glucose production through gluconeogenic and glycogenolytic pathways (Barahona et al. 2024). As shown in Figure 6, the model group exhibited a markedly higher G6P activity (159.84% of the normal group), indicating altered glucose metabolism‐related activity in IR‐HepG2 cells. Moreover, the AO–FO complex exhibited a stronger inhibitory effect on G6P activity than AO or FO alone. These results suggest that the AO–FO complex influences enzymes involved in glucose metabolism. However, the present study did not directly evaluate glucose production, glucose uptake, glycogen synthesis, GLUT expression, or PI3K/Akt signaling; therefore, further investigation is needed to clarify the pathways associated with these effects.

**FIGURE 6:**
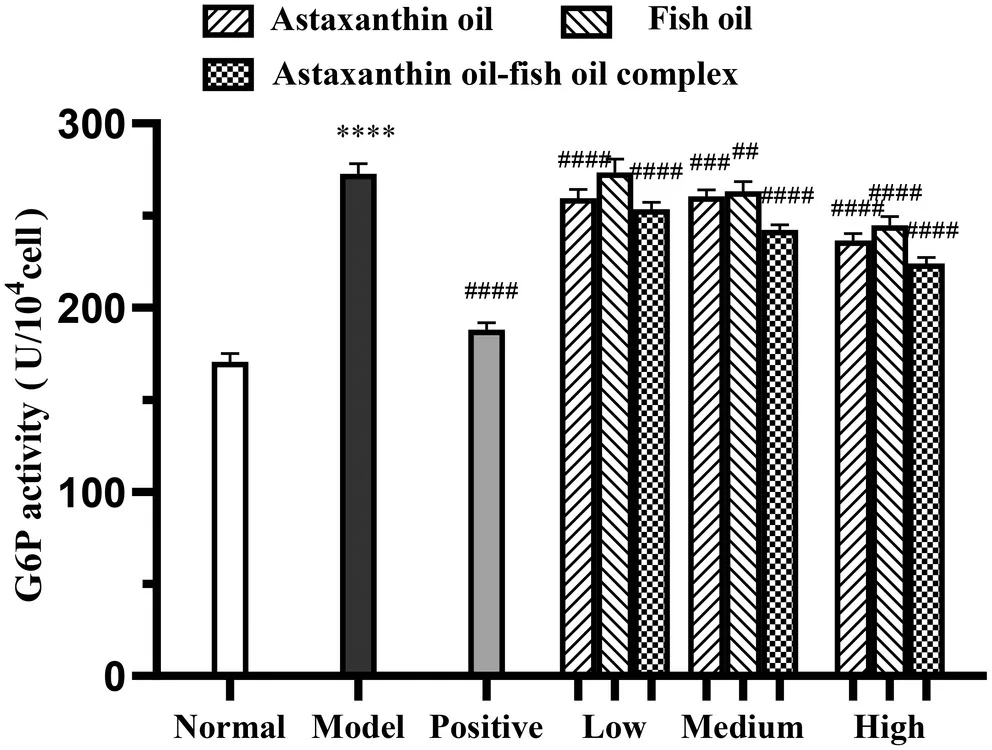
Effect of the test samples on G6P activity in IR‐HepG2 cells. ****p < 0.0001 compared with the normal group, and ##*p* < 0.01 and ###*p* < 0.001, ####*p* < 0.0001 compared with the model group.

#### 3.1.7 Glycogen Detection

Total glycogen level remarkably decreased in the model group (19.179 μg/mg) compared with the normal controls. This finding was consistent with the induced IR phenotype. As illustrated in Figure 7, both AO and AO–FO complex exhibited dose‐dependent improvements in glycogen storage in IR‐HepG2 cells; in contrast, FO alone exhibited significant effects at 600 μg/mL only. Notably, at all tested concentrations (low, medium, high), the AO–FO complex (1:12 ratio) exhibited superior efficacy compared with either component alone. The composite formulation produced significantly higher glycogen accumulation than equivalent concentrations of individual components, indicating a greater combined effect on IR‐related glycogen storage than either component alone. This result was consistent with the promoting effect of AXN and FO on glycogen synthesis in previous studies (Chen et al. 2023).

**FIGURE 7:**
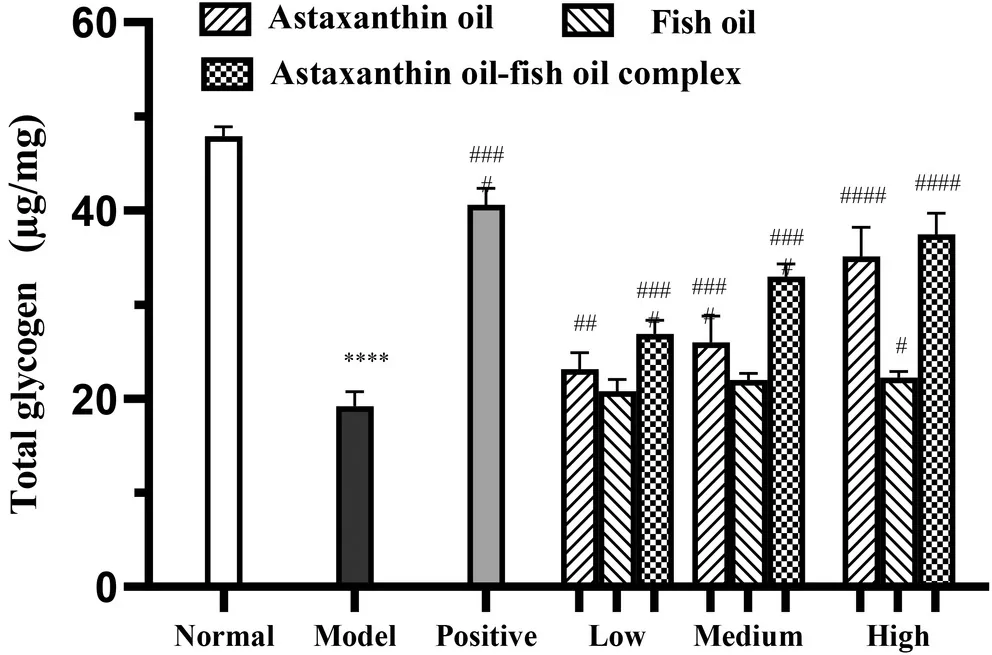
Effect of the test samples on total glycogen accumulation in IR‐HepG2 cells ****p < 0.0001 compared with the normal group, and #*p* < 0.05, ##*p* < 0.01, and ####*p* < 0.0001 compared with the model group.

#### 3.1.8 Detection of Oxidative Stress Indicators

Oxidative stress increases free radicals in the body, resulting in damage to DNA, proteins, lipids, and other substances. Prolonged oxidative stress can result in functional impairments in multiple physiological systems (Chaudhary et al. 2023). SOD and CAT are the most common antioxidant enzymes. Furthermore, MDA indicates the lipid peroxidation level in vivo and indirectly suggests the cell oxidative damage level (Park et al. 2021). As illustrated in Figure 8, SOD activity markedly decreased in the model versus normal groups, while MDA and CAT levels dramatically increased. This confirms that cellular IR is generally accompanied by oxidative stress. Treatment with AO and FO, either individually or in the complex, significantly improved the abovementioned parameters. The AO–FO complex showed more pronounced combined effects. SOD and CAT activities were increased by 43.43% and 10.63%, respectively, in the complex‐M groups (10 μg/mL +120 μg/mL) compared with the model group; however, MDA content decreased by 35.05%. The complex‐H group (50 μg/mL +600 μg/mL) further enhanced these effects, increasing SOD and CAT activities by 66.84% and 21.95%, respectively, and decreasing MDA content by 40.09%. These findings suggest that the AO–FO complex can effectively mitigate oxidative stress by improving antioxidant enzyme activities while reducing lipid peroxidation. This finding is consistent with the strong antioxidant properties of AXN and the function of ω −3 fatty acids in FO in suppressing inflammation (Brown et al. 2019).

**FIGURE 8:**
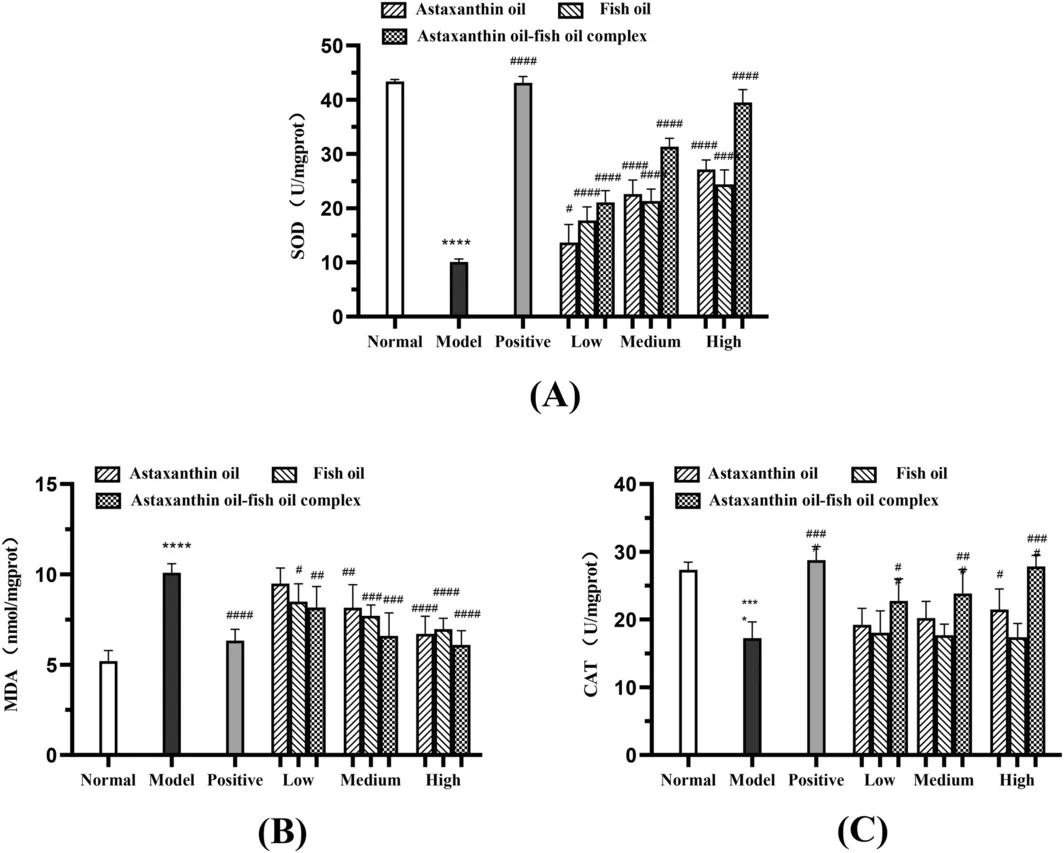
Effect of the test samples on oxidative stress‐related enzymes in IR‐HepG2 cells. (A) SOD activity, (B) MDA content, and (C) CAT activity. ****p < 0.0001 compared with the normal group, and #*p* < 0.05, ##*p* < 0.01, ###*p* < 0.001, and ####*p* < 0.0001 compared with the model group.

### 3.2 Animal Experiments

#### 3.2.1 Detection of Fasting Blood Glucose

As shown in Figure 9, blood glucose levels significantly decreased in the positive versus T2DM model groups, decreasing from 12.68 ± 0.72 mmol/L to 7.45 ± 0.76 mmol/L. Furthermore, blood glucose levels significantly decreased in both AO and FO groups relative to the model group. Notably, the complex‐H group exerted the most pronounced glucose‐lowering effect, reducing blood glucose from 12.76 ± 0.80 mmol/L to 8.81 ± 1.09 mmol/L (*p* < 0.01 vs. model group). The complex‐H group produced a reduction in fasting blood glucose comparable to that in the positive group. These results suggest that the AO–FO complex, particularly at high dosage, effectively ameliorates fasting hyperglycemia in T2DM mice.

**FIGURE 9:**
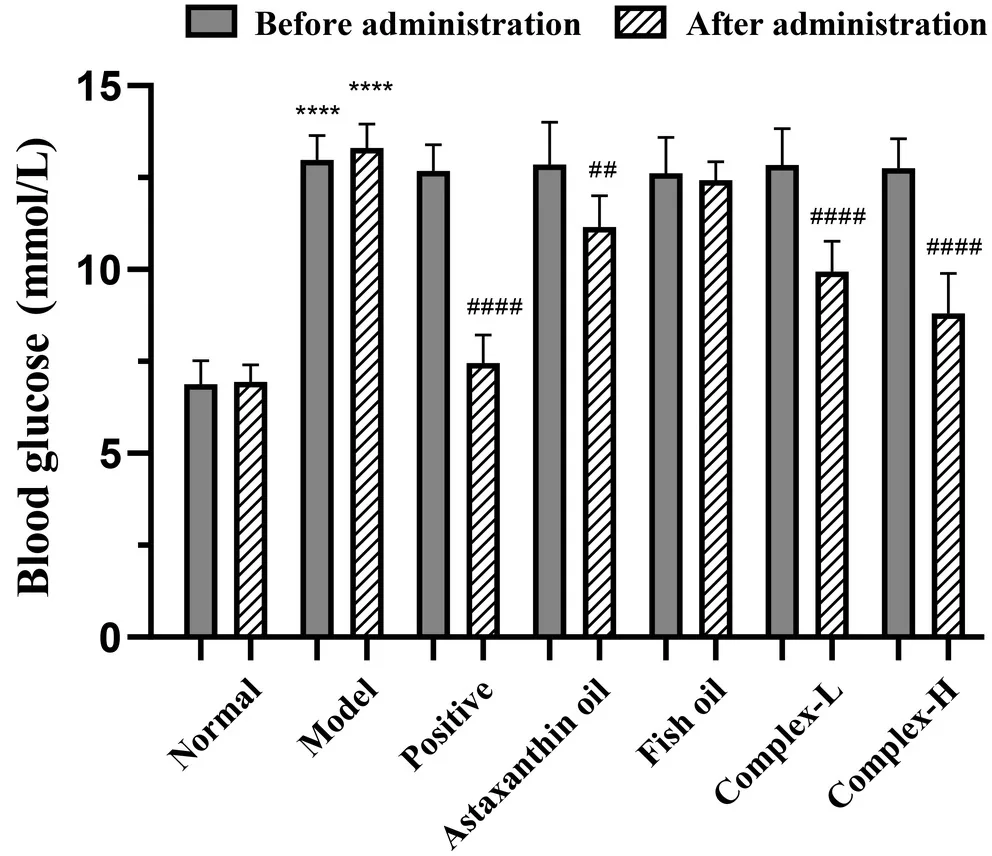
Effect of test samples on fasting blood glucose levels in T2DM mice. ##*p* < 0.01, ####*p* < 0.0001 compared with the model group.

#### 3.2.2 OGTT

OGTT is commonly used to evaluate IR and diagnose DM. It analyzes the circulating glucose clearance capacity of the body following glucose administration after a period of fasting (Liu et al. 2024). Standardized procedures are used to track dynamic blood glucose changes in test animals over time (Liu et al. 2025). As illustrated in Figure 10, blood glucose levels continued to increase at 30 min and reached the peak at 60 min after oral glucose ingestion in the model and FO groups. In contrast, blood glucose levels reached the peak at 30 min after oral glucose ingestion in the other groups. The FO group showed similar changes in blood glucose levels to the model group. The blood glucose levels decreased after 60 min of oral glucose ingestion in the other groups. The decreasing trend was the most obvious in the complex‐H group. Blood glucose levels consistently decreased in both complex‐L and complex‐H groups relative to AO and FO groups, indicating that the AO‐FO complex efficiently promotes the hypoglycemic efficacy of AO.

**FIGURE 10:**
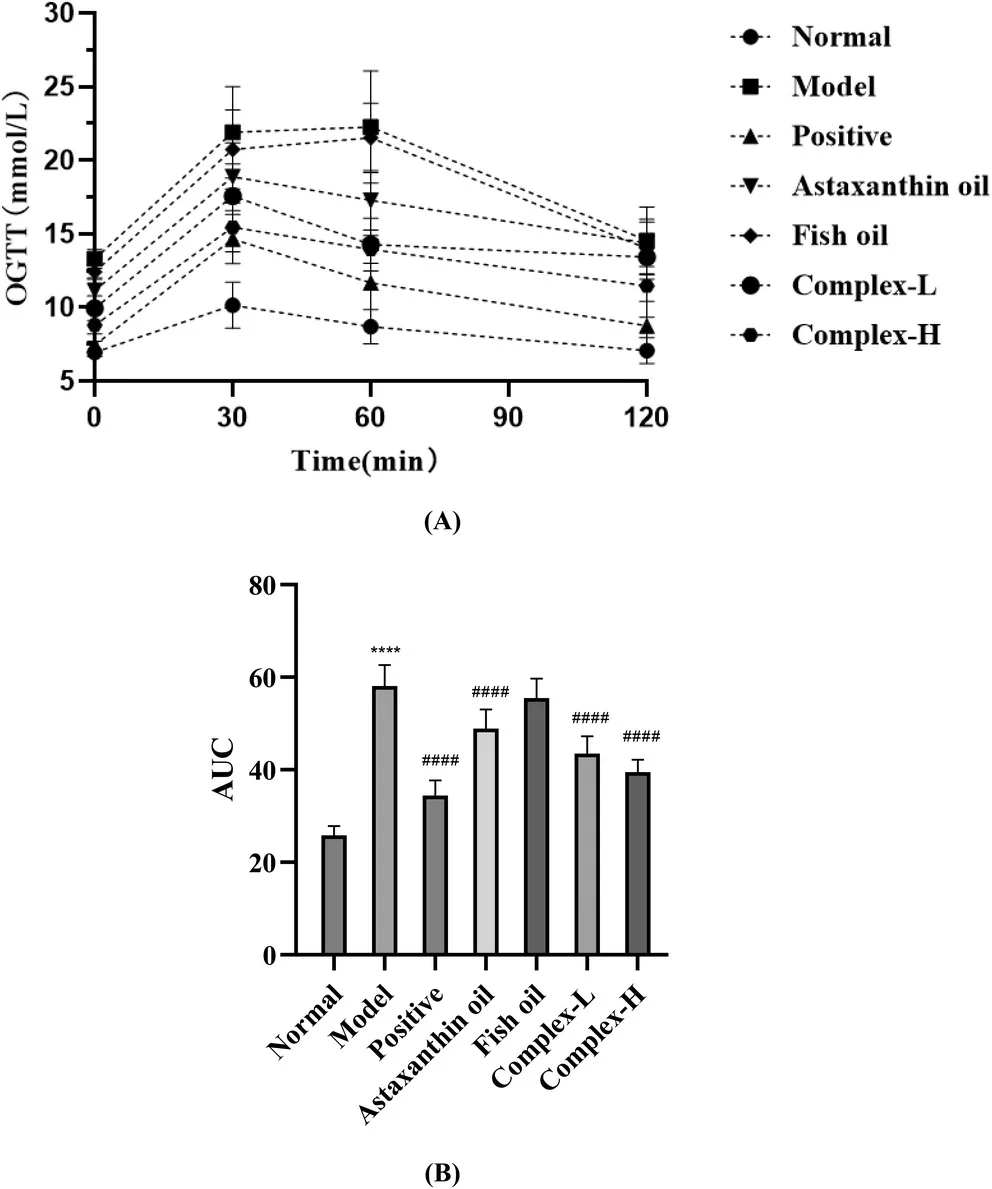
Effect of the test samples on OGTT findings in T2DM mice (A) and the area under the glucose tolerance test curve (B). ****p < 0.0001 compared with the normal group, and ####*p* < 0.0001 compared with the model group.

From Figure 11, the model mice exhibited markedly higher serum ALT and AST levels than their normal counterparts (*p* < 0.01), indicating evident liver injury. Concurrently, this group exhibited significantly increased LDL‐C, TC, and TG contents, as well as a reduced HDL‐C level (*p* < 0.01), confirming that the T2DM mouse model with concomitant dyslipidemia was successfully established.

**FIGURE 11:**
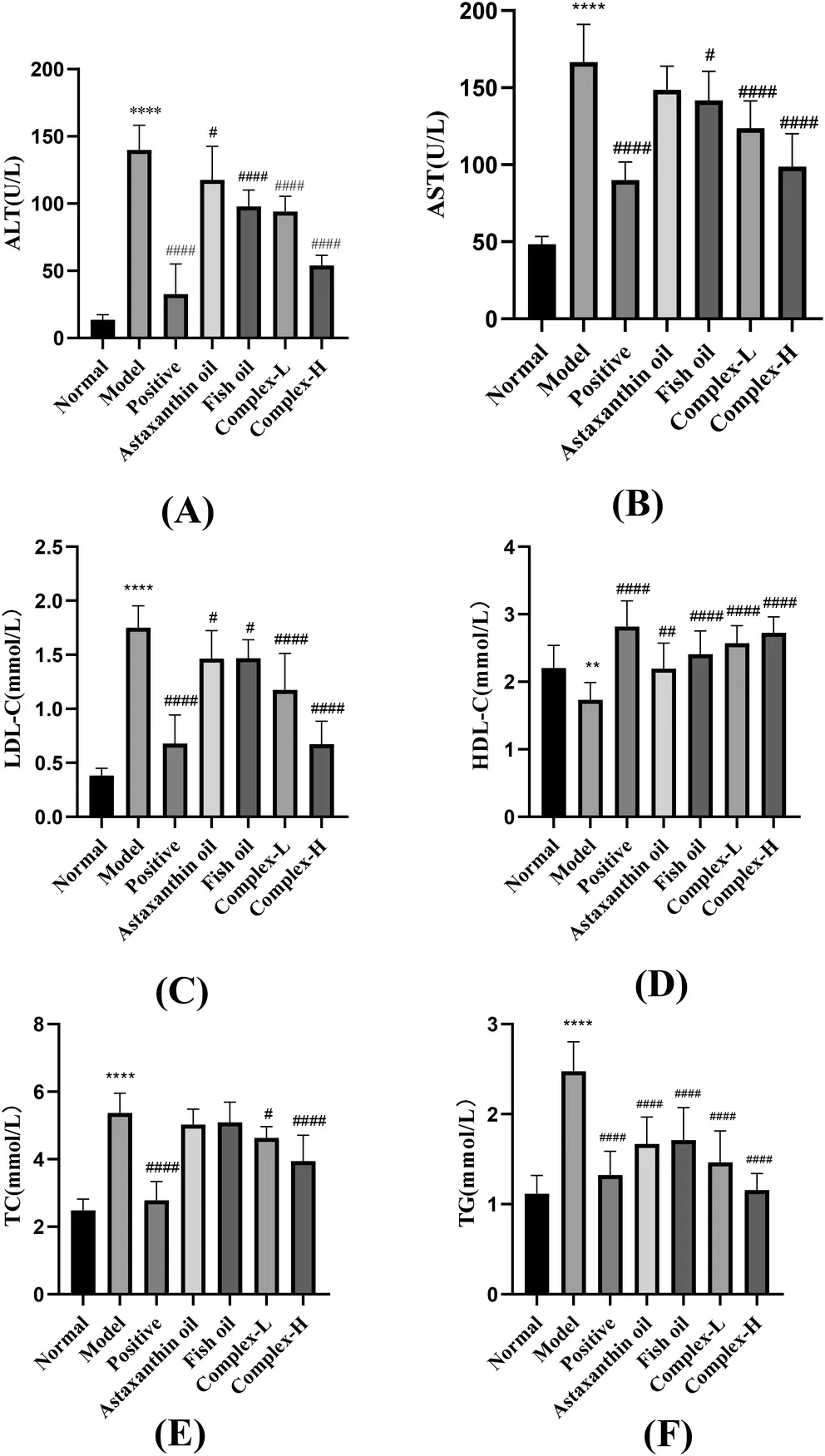
Effects of the test samples on serum biochemical indices. (A) ALT content, (B) AST content, (C) LDL‐C content, (D) HDL‐C content, (E) TC content, (F) TG content. ****p < 0.0001 compared with the normal group, and #*p* < 0.05, ##*p* < 0.01, ###*p* < 0.001, and ####*p* < 0.0001 compared with the model group.

Serum biochemical analysis revealed that AO, FO, and their complex exerted significant regulatory effects on hepatic function markers (ALT and AST) and lipid parameters (HDL‐C, LDL‐C, TG, TC) among T2DM mice. ALT and AST levels were reduced in both AO and FO versus model groups. These effects were more pronounced in the complex groups (*p* < 0.01), particularly in the complex‐H group, which exhibited the greatest reduction (approximately 40% decrease versus the model group). This indicates that the combined treatment was associated with greater improvement in liver injury markers, particularly in the high‐dose group. Concerning lipid profiles, LDL‐C, TC, and TG contents decreased, while HDL‐C contents elevated in the AO and FO groups versus the model group. The complex groups exhibited more significant effects, particularly the high‐dose composite group, which exerted the most remarkable lipid‐modulating effects (with LDL‐C, TC, and TG decreasing by 61.6%, 26.5%, and 53.3%, respectively, and HDL‐C increasing by 57.2%). Collectively, these results suggest that the combination produced greater improvements in dyslipidemia and lipid metabolism in mice with T2DM.

In conclusion, AO plus FO improved serum biomarkers in T2DM mice by reducing ALT/AST levels (indicating liver protection) and regulating lipid profile (decreasing LDL‐C/TC/TG and increasing HDL‐C), with the high‐dose group showing the greatest overall effect.

#### 3.2.3 Detection of Oxidative Stress Indicators in Liver Tissue

As illustrated in Figure 12, SOD and CAT activities and GSH content apparently decreased in the liver of model mice relative to normal mice. Liver MDA contents apparently increased (approximately 50%) in model mice relative to their normal counterparts. These results indicate the successful establishment of the animal model. After treatment, SOD and CAT activities and GSH content remarkably increased among diverse treatment groups. The complex‐H group exhibited the most significant enhancement of hepatic GSH levels and CAT activity, showing elevations of 180.92% and 66.67%, respectively, compared with the model group. After treatment, MDA contents apparently decreased among diverse treatment groups, with the complex‐H group exhibiting the most significant decrease in MDA level.

**FIGURE 12:**
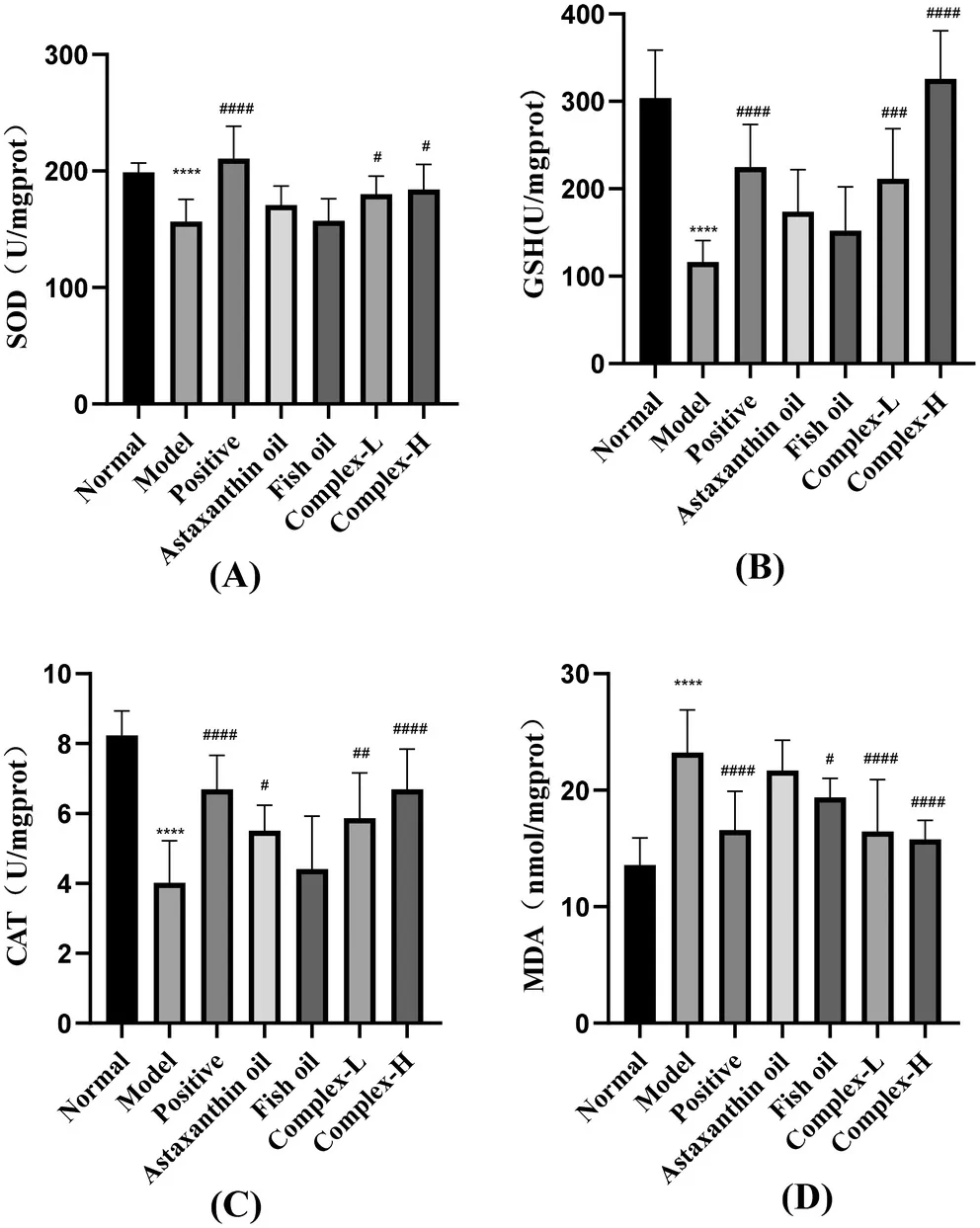
Effect of the test samples on oxidative stress enzymes in T2DM mice. (A) SOD activity, (B) GSH activity. (C) CAT activity. (D) MDA content. ****p < 0.0001 compared with the normal group, and #*p* < 0.05, ##*p* < 0.01, ###*p* < 0.001, and ####*p* < 0.0001 compared with the model group.

#### 3.2.4 Histopathological Examination

As illustrated in Figure 13A, liver histopathological analysis illustrated distinct differences across various groups. In the normal group (a), the liver tissue was healthy, with well‐defined liver lobules and neatly arranged hepatocytes around the central vein, indicating no signs of fat accumulation or inflammation. However, the model group (b) exhibited significant liver damage, characterized by a disordered liver lobule structure, large vacuoles around the central vein, irregular hepatocyte arrangement, and evident fat degeneration and inflammatory cell infiltration. The positive group (c) exhibited some improvement, with more regular liver lobules and decreased inflammatory cell infiltration. Both the AO (d) and FO (e) groups exhibited vacuole reduction and improved hepatocyte arrangement relative to the model group (b). The complex‐L group (f) further enhanced these improvements, with even more regular liver lobules and decreased fatty degeneration. Notably, the complex‐H group (g) exhibited the most significant improvement, with the liver tissue structure closely resembling that of the normal group (a), indicating a greater protective effect of the combined treatment on the liver.

**FIGURE 13:**
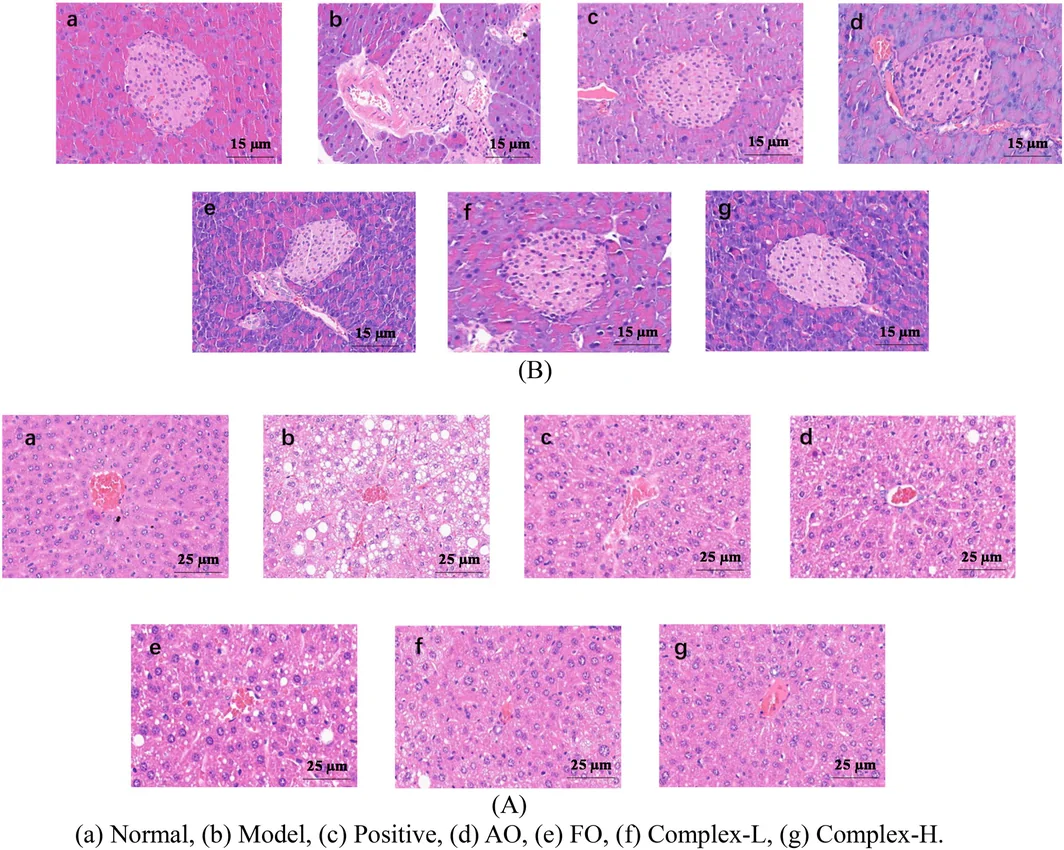
Photomicrographs of pathological tissue sections of mouse liver and pancreas. (A) Mouse liver; (B) Mouse pancreas. (a) Normal, (b) Model, (c) Positive, (d) AO, (e) FO, (f) Complex‐L, (g) Complex‐H.

Histopathological analysis of the pancreatic tissue illustrated distinct differences across diverse groups (Figure 13B). The normal group (a) exhibited intact pancreatic tissue with well‐defined islets and regular boundaries. The model group (b) exhibited severe pancreatic damage, including irregular islet shape, blurred boundaries, decreased cell number, and signs of vacuolization and inflammation. Metformin treatment (c) improved islet shape and clarity. The AO (d) and FO (e) groups exhibited better islet structure and less vacuolization than the model group (b). The complex‐L group (f) further enhanced these improvements. The complex‐H group (g) exhibited the most significant recovery, with the pancreatic tissue structure closely resembling that of the normal group, indicating a greater protective effect of the AO–FO complex.

## 4 Conclusions

This study evaluated the hypoglycemic potential of AO, FO, and the AO–FO complex using both cellular and animal models. In HepG2 cells, the AO–FO complex exhibited no cytotoxicity at 1–200 μg/mL, enhanced glucose consumption, was associated with changes in glucose metabolism‐related indices, increased cellular glycogen content, and decreased oxidative stress. In T2DM mice, the AO–FO complex reduced blood glucose levels, improved lipid profiles, and mitigated liver injury, with histopathological findings supporting improvements in liver and pancreatic tissue morphology. Collectively, the AO–FO complex showed greater hypoglycemic activity than either component alone under the tested conditions. However, because the IR‐HepG2 model has inherent limitations in fully representing the complex pathophysiology of insulin resistance in vivo, these findings should be interpreted with caution. Overall, these findings suggest that the AO–FO complex has potential hypoglycemic activity and may contribute to improvements in glucose and lipid metabolism. Further studies using more physiological relevant models are warranted to validate these findings and to elucidate the underlying mechanisms, including the potential involvement of glucose uptake, insulin signaling, and other related pathways in mediating these effects.

## Author Contributions

**Zhichao Lin:** data curation, methodology, validation, writing – original draft, visualization. **Jianlin He:** conceptualization, methodology, supervision. **Xiaomin Xi:** formal analysis, writing – review and editing. **Yiping Zhang:** project administration, funding acquisition, resources. **Qingqing Le:** data curation. **Yaqi Guo:** data curation. **Hua Fang:** data curation. **Wenhui Jin:** formal analysis. **Hui Chen:** resources. **Sijin Chen:** formal analysis. **Weizhu Chen:** conceptualization, methodology, writing – original draft, writing – review and editing, funding acquisition, investigation.

## Funding

This work was supported by Fujian Provincial Science and Technology Planning Project of China, 2023N0035, 2024Y0077. Xiamen Marine and Fishery Development Special Fund of China, 23CZP009HJ11. The Fund of the Technology Innovation Center for Exploitation of Marine Biological Resources, MNR, TICMBR202303.

## Conflicts of Interest

The authors declare no conflicts of interest.

## Data Availability

The data that support the findings of this study are available on request from the corresponding author. The data are not publicly available due to privacy or ethical restrictions.
